# A Forty-Year Analysis of the Literature on *Babesia* Infection (1982–2022): A Systematic Bibliometric Approach

**DOI:** 10.3390/ijerph20126156

**Published:** 2023-06-16

**Authors:** Samson Anjikwi Malgwi, Ropo Ebenezer Ogunsakin, Abolade David Oladepo, Matthew Adekunle Adeleke, Moses Okpeku

**Affiliations:** 1 Discipline of Genetics, School of Life Sciences, University of Kwa-Zulu Natal, Westville, Durban 4000, South Africa; 2 Biostatistics Unit, Discipline of Public Health Medicine, School of Nursing & Public Health, College of Health Sciences, University of Kwa-Zulu Natal, Durban 4000, South Africa

**Keywords:** babesiosis, bibliometric, cosmopolitan, tick-borne, Web of Science (WoS)

## Abstract

*Babesia* infection is a tick-borne protozoan disease associated with significant veterinary, economic, and medical importance. This infection affects many hosts, ranging from wild to domestic animals and including man. All vertebrates serve as potential carriers due to the huge diversity of the species. Babesiosis has been associated with severe economic loss in livestock production, especially in cattle farming, and is also a major public health concern in man, which could be fatal. The infection is usually opportunistic, ranging from asymptomatic to symptomatic, usually in immunocompromised subjects or under conditions of stressful management. This study was designed to uncover trends in relation to publication growth and further explore research output regarding babesiosis from data indexed in the WoS. The WoS is the only platform used to map publications on *Babesia* infection. The search term “babesiosis” or “*Babesia* infection” was used to extract articles published across the study period from 1982 to 2022. The inclusion criteria were restricted to only articles for the analysis. The results from the search query showed that a total of 3763 articles were published during the study period with an average of 91.70 ± 43.87 articles annually and an average total citation (n = 1874.8). An annual growth rate of 2.5% was recorded during the study period. The year 2021 had the highest number of published articles (n = 193, 5.1%) and citations (n = 7039). The analysis of the most relevant keywords and titles showed that infection (n = 606, 16.1%), babesiosis (n = 444, 11.7%), and Babesia (n = 1302, 16%) were the most relevant keyword plus (ID), author keyword (DE), and title, respectively. The common conceptual framework analysis through K-means clustering showed two clusters comprising 4 and 41 elements, respectively. The United States of America is the top-performing country in terms of article production (n = 707, 20.8%) and the leading funder for babesiosis research, with two of its agencies ranked at the top. These are the Department of Health and Human Services (n = 254, 6.7%) and the National Institute of Health (n= 238,6.3%). Igarashi I. is the top-performing author (n = 231, 6.1%), while *Veterinary Parasitology* is ranked the top journal (n = 393, 10.4%) in terms of babesiosis publications. Overall, an increase in publications was observed in the study period, with significant output from developed nations.

## 1. Introduction

Tick-borne pathogens are often known to pose a serious threat to a wide range of hosts, with consequent public health risks because they are known to harbour many pathogens, especially with the growing threat of ticks covering a larger geographic region in the last decade [1]. Ticks serve as vectors in the transmission of various pathogens, such as protozoa, viruses, and bacteria, accounting for most vector-borne diseases across the globe [2]. Babesia is a major protozoan parasite transmitted by ticks. Babesia was first discovered in 1883 when Victor Babes (1888) [3] observed the microorganisms from red blood cell samples obtained from cattle in Romania, which were presented with clinically manifesting haemoglobinuria. Similarly, a few years later, Smith and Kilborne identified an intraerythrocytic organism in the United States of America (USA) as the aetiology of Texas Cattle Fever, with ticks identified as vectors. This disease has ravaged cattle ranchers in the southern part of the country [4]. The manifestation of the infection is often influenced by several intrinsic factors of the host such as genetic factors, age, immunity status, and other concurrent aetiologies. The clinical manifestations of acute infections in hosts include pyrexia, anorexia, anaemia, haemoglobinuria, icterus, and malaise, with the chronic infection being asymptomatic with no clinical manifestation [5].

The aetiology of the infection is largely host-specific. However, few species are known to infect multiple hosts. The parasite infects a wide range of hosts, and species of medical importance infecting animals include *B. ovis*, *B. crassa*, and *B. motassi* in small ruminants [6]; *B. canis*, *B. rossi*, and *B. vogelli* in dogs [7]; *B. bovis*, *B. bigemina* and *B. divergens* in cattle [8]; *B. microti* in rodents [9]; and *B. caballi* in equines [10]. These parasites invade the red blood cell of the host, leading to severe anaemia. The major economic importance of babesiosis is seen adversely in livestock production, especially in bovine babesiosis [11]. These species result in severe economic losses owing to emaciation, a decrease in milk and meat production, and foetal loss due to abortion in the third trimester of pregnancy, leading to a low birthrate [12]. This adversely affects the productivity of the farmers and ultimately decreases income. A key factor is also the increase in the cost of production due to the procurement of drugs for treatment and also acaricides to eliminate the tick vector [13]. The prolong use of acaricides has also been associated with the contamination of meat and milk due to the presence of residues [11]. In severe infection, nervous manifestation such as seizures, paralysis, and respiratory compromise is noticed, and death ensues as a result of shock [14]. Babesiosis serves as an impediment to the improvement in animal traits via cross-breeding and also the restriction of transboundary trade due to quarantine laws [15]. Despite the application of various preventive and control strategies, cases of babesiosis have been reported to increase due to the failure of available live vaccines and acaricidal efficacy [16]. Human babesiosis is regarded as an emerging zoonosis even though the etiological agent is primarily of another definitive host. *B. divergens* and *B. microti* are responsible for the infection in man. The infection may be fatal in splenectomised or immunocompromised subjects [17]. The surge in the number of cases of babesiosis is on the increase in the human population. This increase has been associated with several host–pathogen interactions and environmental factors. The close association between man and animals also plays a key role in transmission, especially animals that are already established as reservoirs of the infection. Human babesiosis has been reported in many parts of the world, such as in Europe with *B. divergens* [18] as the main aetiology and *B. microti* [19] in the United States. *Babesia* is the most common parasite isolated in case of blood transfusion in America. A major concern attributed to asymptomatic infections is the transmission of the infection via transfusion. In Africa, *B. divergens*-like infections have been reported in the Canary Islands, and uncharacterised *Babesia* spp. has been reported in Egypt, South Africa, and Mozambique [20]. Human babesiosis is apparently a subclinical infection but produces malaria-like symptoms in severe cases, especially in older subjects or immunocompromised individuals. Death has been reported, especially in 10% of individuals with *B. microti* infection [18,21]

Several studies exist on the transmission, life cycle, pathogenesis, clinical signs, and symptoms of the infection. Many studies are available on vector control since they are known effective vaccines yet chemotherapeutic agents. Studies have established the impact of the infection on various hosts, which has promoted much research in this field. The application of bibliometric analysis has been extensively used to provide textual analysis on several infectious diseases such as leishmaniasis [22], toxocariasis [23], tuberculosis [24], schistosomiasis [25], Chagas [26], and malaria [27]. However, there is no information regarding analysis involving babesiosis to date. This study aims to uncover the number of articles and citations, top-performing authors, and countries, with a co-occurrence network of keywords over a 40-year period using the Web of Science database.

Thus, there is a need for a novel approach to improve the understanding in a comprehensive way that can increase the benefits of the work of young researchers for a broader population. The different study methods, which only assist in understanding a portion, need to be assembled and brought under the same ridge to present a holistic view. Hence, this paper aims to map and identify the conception and evolutions of babesiosis through the technique of systematic bibliometric analysis. Bibliometrics and visualisation have been used to identify the occurrence of diseases, which is a technique for scientific evaluation [28]. They can be used systematically to analyse publications in terms of author impact, citations, and geographic locations. These tools were deployed to study essential features of the study subject built on print and also facilitated quantitative and qualitative data appraisal. In summary, this study provides an overview of textual analysis of studies in the literature on babesiosis and identifies key research strongholds and collaborative links and areas of future research.

## 2. Materials and Methods

### 2.1. Database and Search Technique

A prerequisite requirement for any bibliometric study is the identification of an appropriate database, an efficient search strategy, and a swift process of document retrieval. In this present study, the Web of Science (WoS) database was exclusively. The WoS database is the most widely used in the literature and citations owing to its reliability, validity, and accuracy [29,30,31]. It is remarkable that the emergence of scientific databases such as Scopus and Web of Science (WoS) has made acquiring large volumes of bibliometric data comparatively easy. This is one of the largest and most comprehensive databases. The WoS is being maintained by Clarivate, and its core collection spans various disciplines and documents. It has an estimated over 11,000 authoritative journals that cut across various fields. In the current study, data analyses were based on the information collected from the WoS. The document type search was limited to only articles, and the search method was title search. 

A valid and reliable comprehensive data strategy was developed for the retrieval of relevant literature that would minimise errors in order to avoid false-positive outcomes. To achieve this, many publications concerning bibliometric analysis were exclusively studied to perfect a reliable search query for babesiosis [32,33,34,35]. The data for this study were obtained from the WoS core collection on 1 May 2022. The search query involved a title search using the keywords “babesiosis” or “*Babesia*”. An inclusive search term babesi* was used for the search query. The study period was between 1 January 1982 and 31 December 2022. The search was refined for only articles to be selected as the type of document. Finally, a total of 3763 articles were exported and utilised for further analysis. The search query can be presented as follows: 3763 results; babesi*(title); refined by document types: article; timespan: 1 January 1982 to 31 December 2022 (index date). All relevant data that would facilitate the analysis were exported, including but not restricted to annual publication growth, total article citations, most relevant keywords, funding agencies, top-performing countries, and authors. 

**Figure 1:**
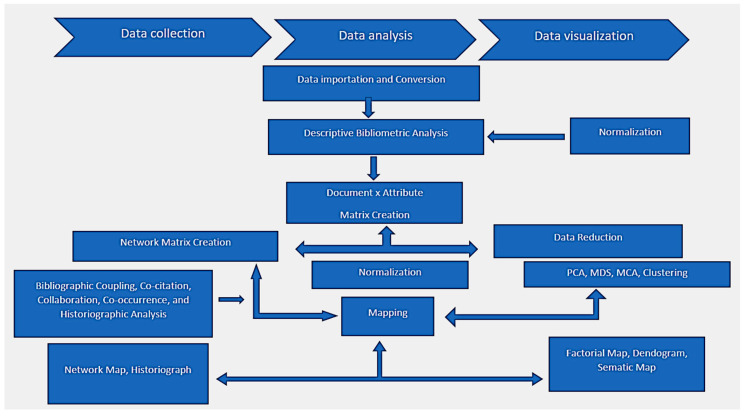
Flowchart of bibliometric processing of information (source [37]). NB: PCA, principal component analysis; MCA, multiple correspondence analysis; MDS, multidimensional scaling.

The final phase of analysis involved only articles. This is because articles are complete research with definitive results. Data downloaded for each article comprise detailed information from the articles. The downloaded data were exported in a BibTex format and were further compressed in a zipped folder and later imported into the biblioshiny interphase. Data analysis involved the use of the R tool with biblioshiny interphase (4.1.3) and MS Excel (v 16.0). It is important to highlight phases that help in understanding quantitative and qualitative bibliometric analysis, as graphically represented in Figure 1. This study presents the results from bibliometric analyses based on the publication records of *Babesia* infection research for the period 1982–2022. This study aimed to assess the *Babesia* infection research output by mapping the type and amount of *Babesia* infection research conducted globally since 1982. The mapping also describes affiliations, the level of collaborations, and the sources of funding for [36] research globally.

### 2.2. Bibliometric Research Methodology

Bibliometric analysis can be divided mainly into two subcategories: science mapping and performance analysis. Science mapping is doctored on the principle of relationships that exist between research components [36,38], while performance analysis relies on contribution towards research components. In science mapping, the analysis involves either intellectual or structural networks that exist among research components. The approach for science mapping involves co-citation analysis, citation analysis, co-word analysis, and bibliographic coupling. The efficient utilisation of these tools in combination with network analysis is vital in presenting both intellectual and structural relationships in a desired research field [39,40]. Each of these analyses has its specific usage, unit of analysis, and data requirement [41]. Performance analysis, in a nutshell, scrutinises the various contributions of research components in a desired field of study [38]. The information provided by such analysis is descriptive, a key distinguishing feature of bibliometric studies [42]. This type of analysis could include different research components such as authors, countries, journals, and institutions. Commonly used measures in performance analysis in most bibliometric studies include citations per year, number of publications, single or multiple authored publications, average citations, and h-index. Apart from these analyses, a few more enrichment tools exist to further complement and improve data analysis. These tools include clustering [43], network metrics [44], and visualisation [45,46].

To avoid errors and ensure the reliability of the study, author keywords, keyword plus, and authors’ names were extracted and screened more than once as different sets (A and B). Proper checking was carried out for spelling errors. Normalisation was achieved when |*A* ∩ *B*| ≡ |*A* ∪ *B*|. In keyword or title analysis, multiple occurrences of a particular word were regarded as one. Keywords comprising author keywords (DE) and keyword plus (ID) were analysed as the overlap comprising these two sets (*DE* ∩ *ID*||). A Venn diagram was used to facilitate the analysis. The relevant functions of the bibliometrix R package were used to analyse author productivity in terms of the h-index. A bibliometric two-way tripartite model (Articles × Attributes) was used in bibliometric networks and bibliographic coupling. The Fruchterman model (force-directed algorithms) was used to create a graphic model of all networks, using the network plot function of the bibliometrix R package. The networks were standardised using various indices such as inclusion index, proximity index, and similarity index. The conceptual structure package was used to perform K-means clustering of keywords. 

The bibliometrix R package and the biblioshiny software tool were used to perform the analysis in this paper [47]. This software allowed us to perform various types of analyses such as citation analysis, co-citation analysis, bibliographic coupling, co-word analysis, and co-authorship analysis. Co-citation analysis involved the analysis of the relationships among cited publications to understand the development of foundational themes in a research field (the unit of analysis was articles). On the other hand, the bibliographic coupling technique was used to analyse the relationships among citing publications to understand the periodical or present development of themes in a research field.

## 3. Results and Discussion

In the study period between 1982 and 2022, a total of 3763 articles were published by 10,557 authors across 512 journals. Detailed information regarding the presentation of the exported data for the analysis is shown in Table 1. In terms of author/article analysis, values of 0.356, 2.81, and 5.52 were obtained for an article per author, author per article, and co-author per article, respectively. Notably, 120 (1.43%) authors published articles as single authors, while 10,437 (98.9%) authors published articles as multiple authors, with a collaborative index of 2.89. 

### 3.1. Publication Growth

**Figure 2:**
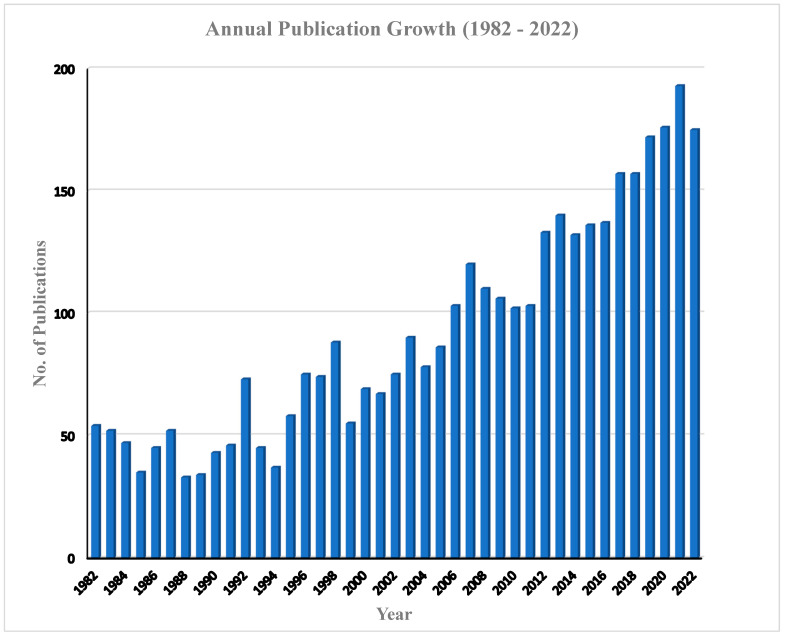
Growth of publications (articles) on babesiosis research (1982–2022).

**Figure 3:**
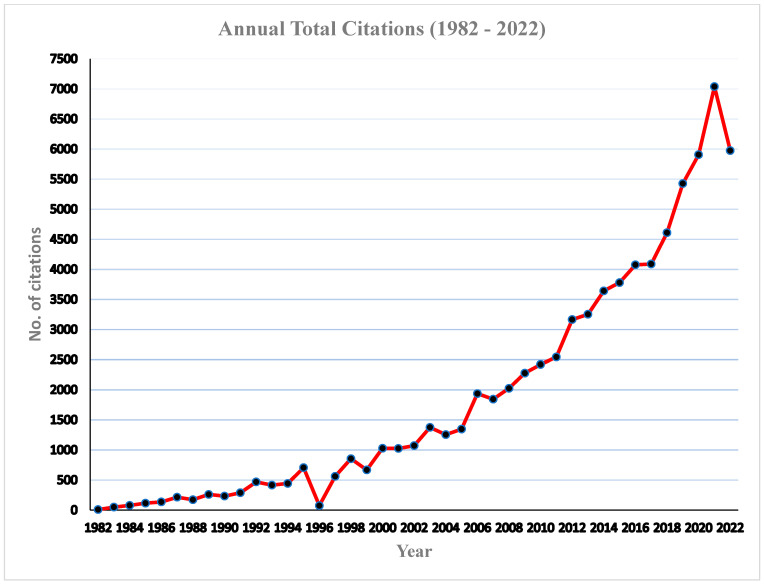
Annual total citations of articles on babesiosis (1982–2022).

The annual publications of articles on babesiosis during the study period (1982–2022) are presented in Figure 2. There was a steady increase in the number of publications, with mean values of 91.70 ± 43.87, and an annual growth of 2.5% was observed during the study period. The peak period of article production in the research field of babesiosis was recorded in 2021, with 193 (5.1%) articles, while the least number of articles was recorded in 1988, with only 33 (0.87) articles. Even though babesiosis, unlike other diseases, is a neglected tropical disease, few factors have been associated with the steady increase in the number of publications, including the devastating effect of the infection, especially in livestock, leading to severe economic loss by farmers [13,48]. Secondly, there has been an increase in the number of human babesiosis infections, especially in the USA, Europe, and Asia, with few cases, although not clinical, recorded in Africa and South America [49,50]. This has led the WHO to classify this infection as an emerging zoonosis [51]. Several scholars have attributed the underreporting of human babesiosis in Africa to its similarity to the clinical manifestation of infection with malaria, especially where malaria is endemic, there is poor disease surveillance, and limited funding is allocated for research. The total annual citations is presented in Figure 3. A steady increase in the number of total citation was clearly observed, with an average of 1874.8 citations per year; however, the maximum number of citation was recorded in the year 2021, with a total of 7039 citations. Similarly, the highest number of publication was in the same year.

### 3.2. Co-Word Analysis

#### 3.2.1. Analysis of Titles

**Figure 4:**
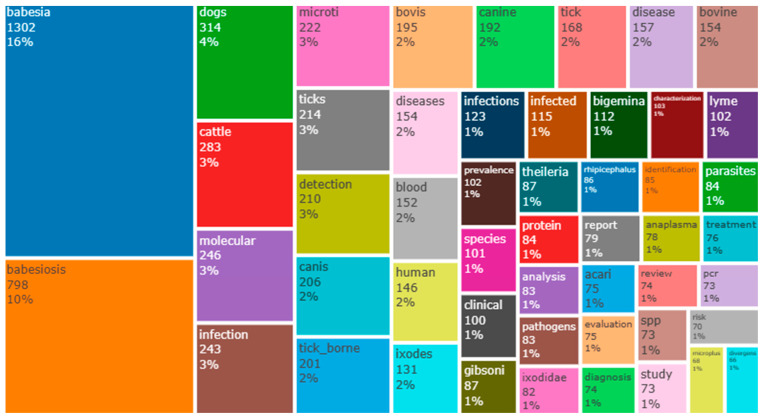
Tree-map analysis of words used on the title of research in the literature on babesiosis.

This analysis was performed using a tree map of the most frequently used words in the title of research on *Babesia* infection, and the results are shown in Figure 4. *Babesia* was the word with the highest frequency of 1302 (16%). Babesiosis, dogs, and cattle were among the top four most frequently used words in the title of research on the study area, with frequencies of 798 (10%), 314 (4%), and 283 (3%), respectively.

#### 3.2.2. Analysis of Most Relevant Keywords

The analysis of the most relevant keyword plus (ID) and author keywords (DE) is presented in Table 2. The most relevant keyword plus was infection, with a frequency of 606 (16.1%), while babesiosis was the most common author keyword (DE), with a frequency of 444 (11.7%). However, six words were common to both keyword plus and author keywords: diagnosis, ticks, PCR, cattle, dog, and *Theileria*. In keyword analysis, a total of 14 words were unique (infection, identification, *Bovis*, prevalence, transmission, *plasmodium falciparum*, diagnosis, *microti*, *bigemina*, parasites, blood expression, antibodies, molecular characterisation, and malaria). Similarly, 14 words were also unique to author keywords (babesiosis, *Babesia*, *Babesia canis*, *Babesia gibsoni*, *Babesia caballi*, ELISA, *Babesia divergens*, Canine babesiosis, epidemiology, and *Babesia* spp.). It is important to note that the most relevant unique author keywords were centred on the aetiology of babesiosis across various host ranges, epidemiology, and diagnosis of the infection, while the most relevant unique keyword plus mainly involved blood sample collection, the identification of these parasites using various diagnostic approaches, transmission, and the similarity of this infection with plasmodium falciparum, the etiological agent of human malaria. Keyword plus terms related to the diagnosis of babesiosis included identification (n = 282, 7.4%) and molecular characterisation (n = 147, 3.9%), while in author keyword (DE), ELISA (n = 84, 2.2%) was associated with the diagnosis. In terms of co-infections, *Plasmodium falciparum* (n = 231, 6.1%) in keyword plus (ID) and *Theileria equi* (n = 67, 1.7%) in author keywords were related to babesiosis research.

#### 3.2.3. Analysis of Thematic Evolution

**Figure 5:**
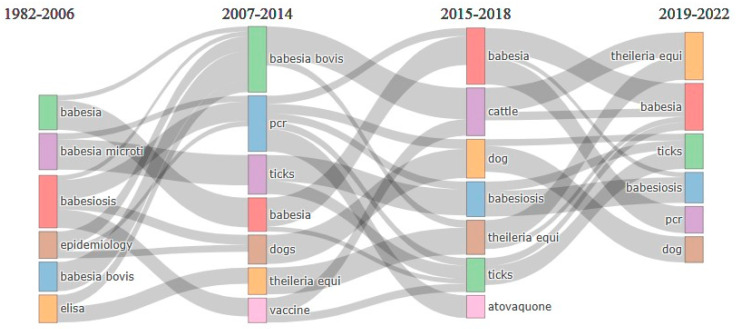
Analysis of thematic evolution of keywords in research articles on babesiosis from 1982 to 2022. Note: Colour helps in the identification of different research themes. Thicker lines indicate higher significance between the two periods.

Bibliometrics refers to the quantitative evaluation of the occurrence of certain events in the scientific literature, as opposed to the analysis and interpretation of the content of the literature. In the context of this study, bibliometrics was employed to condense the most illuminating outcomes of a collection of bibliographic papers. This analysis provides information associated with evolutionary trends and changes across different subperiods [38,52]. To identify evolutionary changes, a thematic analysis for *Babesia* infection was carried out for the period 1982–2022. A Sankey diagram was used to visualise and understand the analysis. This diagram comprises a node that represents a particular subject, and its size is determined by the frequency of the keyword. A line shows the connection between two different nodes. The width is determined by several keywords. The study period (1982–2022) was divided into five categories: 1982–2006, 2007–2014, 2015–2018, and 2019–2022, as shown in Figure 5.

Thematic evolution analysis was carried out using author keywords. From 1982 to 2006, which was the first category, five research areas underwent thematic evolution: Babesia, *Babesia microti*, babesiosis, epidemiology, *Babesia bovis*, and ELISA. At this phase, primary information about the pathogen, most especially *Babesia microti* affecting humans and *B. bovis* having a devastating effect on cattle, was documented, identifying the epidemiology of these infections. However, the second phase (2007–2014) showed the emergence of five new thematic areas, namely PCR, ticks, dog, *Theileria equi*, and vaccine, while *Babesia* and *Babesia bovis* remained as primary themes. The increase in the number of thematic areas suggests an underlying increase in the number of articles on *Babesia* infection and therefore indicates that thematic evolution has been ongoing.

Serology and microscopy (Giemsa-stained smear) were used for diagnosis during the early period. However, with advancements in technology and knowledge, the limitations of these techniques, such as time consumption, lack of specificity, difficult protocols, and failure to distinguish between previous and current exposure, became their major drawback [53]. Serology, especially enzyme-linked immunosorbent assay (ELISA), has been performed predominantly in numerous studies to detect antibodies in Babesia infection [54,55,56,57]. Molecular approaches such as PCR (polymerase chain reaction) are currently the best techniques for the detection of *Babesia* infection. Real-time reverse transcription polymerase chain reaction (RT-PCR) and next-generation sequencing (NGS) are presently the best diagnostic approaches and have been used by several researchers [58,59,60,61]. Vaccine development became a challenge until the development of molecular technologies, which provided the template for its development. Before this advancement in development, animal vaccines, in particular, consisted of the subinoculation of attenuated (weak) parasites from asymptomatic carriers to susceptible animals. This is commonly referred to as premunition, which is a form of immunoprophylaxis. This type of vaccination has several limitations, including the presence of contaminants in the blood vaccine (e.g., bovine leucosis virus [62]) and emerging pathogens, variation in virulence, failure to induce premunition, and dependence on liquid nitrogen for storage [63]. This led to the development of modern-day vaccines with the use of current molecular approaches to produce vaccines through technologically supported immunoprophylaxis procedures that have been tried for several years. Some researchers in Australia have tried this approach using passaged *Babesia* strains with reduced virulence, from which the blood is used as a vaccine [64]. However, some limitations mentioned earlier such as the availability of cold chains and the presence of contaminants are still applicable. Despite the application of molecular approaches, recent reports suggest that effective immunogens against the infection are yet to be achieved, while only live vaccines have shown the ability to protect against the disease in immunised animals when infected with the virulent pathogen [65]. In the third period (2015–2018), *Babesia*, cattle, dogs, babesiosis, *Theileria equi,* ticks, and atovaquone evolved in the thematic evolution. Similarly, in the fourth period (2019–2022), *Theileria equi*, *Babesia*, ticks, babesiosis, PCR, and dog were observed as thematic patterns. These results showed that the development of efficacious drugs for the treatment of the infection became a focus for the researchers, which led to the discovery of atovaquone. The initial treatment of the infection involves the use of clindamycin and quinine compounds, especially in human babesiosis. However, due to the adverse reaction of these drugs, further research was conducted to develop a drug with a mild reaction [66]. In some cases, treatment with quinine had to be stopped as a result of severe adverse reactions. Due to adverse reactions, atovaquone became the drug of choice for chemotherapy, especially in combination with azithromycin, in moderate-to-severe infections [67,68]. New drugs are still evolving, and further research is underway to identify new drug-binding sites of this parasite. Cysteine proteases (CPs) are known as therapeutic targets for several apicomplexan parasites. In *Babesia*, it has been established through research that CPs reduce in vitro invasions of erythrocytes and the replication/growth of *Babesia bovis*. These are considered potential chemotherapeutic agents upon the conclusion of molecular mechanisms of these CPs on *Babesia* species and so many other targets in the post-genomic era [69]. Through thematic evolution, PCR and ticks increasingly became the focus of research, and as a result, further research on ticks using this approach led to the identification of emerging tick-borne diseases, which were hitherto not discovered. All this information has been uncovered due to advancements in technology, which provided a better overall understanding of the infection [70].

### 3.3. Analysis of Authors

The analysis involved 10,560 authors, who published a total of 3763 articles across the study period. Of these 10,560 authors, 120 authors published as single authors, while 10,437 published articles as co-authors (multiple authors). As shown in Table 3, the top ten authors are Igarashi I., Xuan X., Yokoyama N., Li Y., Yin H., Luo J., Guan G., Banneth G., Fujisaki K., and Brown W.C., with 231, 139, 130, 67, 65, 57, 47, 45, and 44 articles, respectively. Igarashi I. is a leading author in terms of the number of published articles, average citation, h-index, and g-index, respectively. For example, Igarashi [71] co-authored a paper that focused on the diagnosis of the infection using a multiplex loop-mediated isothermal amplification method, published in the *Journal of Microbiology Methods*, with over 200 citations. This technique is convenient and reliable and can be used for the detection of bovine *Babesia* parasites simultaneously. Another article he co-authored involves the development of new molecular techniques for the diagnosis of equine babesiosis, which has over 180 citations and is published in *Veterinary Parasitology* [72]. The proposed technique paved the way for the routine diagnosis of the infection and is used as a tool in epidemiological studies. This author has been publishing articles since 1990.

**Figure 6:**
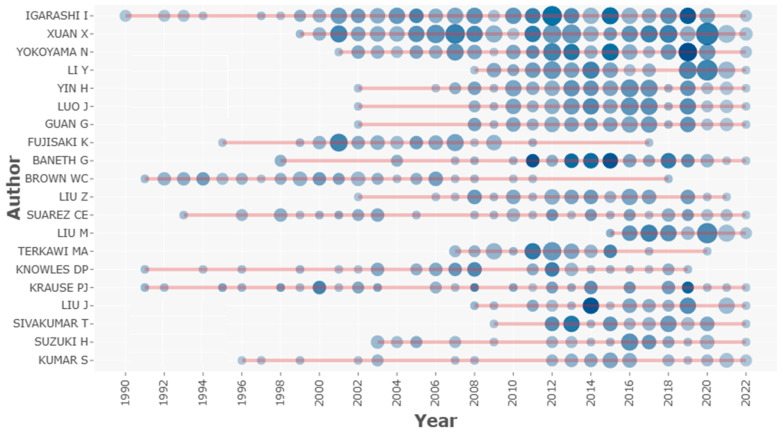
Authors’ productivity on babesiosis. Note: The larger the circle, the higher the number of documents, and the darker the colour, the higher the number of citations.

Similarly, authors’ productivity over time considering the literature on babesiosis is graphically presented in Figure 6. In the year 2012, Igarashi I. had the highest number of publications (11) and mean citations per item (17.82). Another top-rated researcher in this field is Xuan X., with prominent figures regarding the number of publications (139), h-index (23), and g-index (32). In 2021, Xuan X. had the highest number of publications (16) and (19) citations, as graphically represented in Figure 5. Most of his research outputs were published in high-ranking journals in terms of impact factor. A notable example is his contribution to the genetic (transplacental) transmission of *Babesia gibsoni* infections in dogs, published in the *International Journal for Parasitology*, with 138 citations, which provided insight into vertical transmission [73]. The study was able to establish generational transmission via the uterus and not trans-mammary as it was earlier speculated. This was the first confirmatory report on the transplacental transmission of *Babesia* species within a definitive host. A study published in *Veterinary Parasitology*, which centred on the use of both serology and molecular procedures to detect *Babesia* infection in water buffaloes from Thailand, has 125 citations so far [74]. The results of the study showed variation in the prevalence of the infection owing to the sensitivity and specificity of each diagnostic approach used, which was statistically significant. The overall prevalence of *B. bovis* and *B. bigemina* was, respectively, 16.8% and 5.6% using the indirect fluorescent antibody technique (IFAT), 11.2% and 3.6% using nested polymerase chain reaction (nPCR), and 14.7% and 5.9% using the enzyme-linked immunosorbent assay (ELISA). Xuan X. has been contributing to the literature on *Babesia* infections since 1999.

### 3.4. Country Performance

Globally, 138 countries were involved in journal publications on babesiosis between 1982 and 2022 across various continents of the world. Countries with high publications revealed the significance of babesiosis within the study area. The literature related to *Babesia* infection in the top countries is shown in Table 4. North America, Asia, and Europe were the leading contenders in terms of the number of published articles across the various continents. The list of the top 20 countries with high research output included 11 European countries (Poland, Germany, France, Italy, Spain, Turkey, Israel, the Netherlands, Portugal, Switzerland, and the United Kingdom), 4 Asian countries (Japan, China, India, and Iran), 2 South American countries (Brazil and Argentina), 1 North American country (United States of America), and 1 African country (South Africa). The USA had the highest number of articles in the study area of interest, and this is no surprise as the country tops the list in terms of funding for research and the accessibility of such funds. Another key factor is its high level of intranational and multinational collaborations. In terms of countries with the most citations, the USA again topped the list, with a total of 24,155 citations, followed by Japan, with 6295 citations.

### 3.5. Multiple Correspondence Analysis (Conceptual Frame)

**Figure 7:**
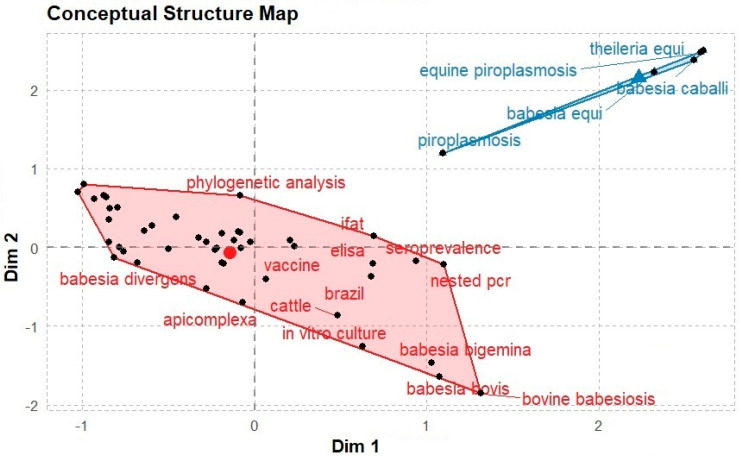
Conceptual frames associated with babesiosis studies (1982−2022)**.** The 3763 articles used for the study showed K-means clustering with two clusters with 41 and 4 elements, highlighting the conceptual frameworks linked to babesiosis. Note: The elements closest to the centre are the essential elements.

The conceptual framework for the data during the study period (1982–2022) was analysed using K-means clustering comprising two clusters of 41 and 4 elements, which are graphically presented in Figure 7. The clusters focused on a vaccine, phylogenetic analysis, cattle, serology (IFAT and ELISA), bovine babesiosis (*B. bigemina*, *B. divergens*, and *B. bovis*), and equine piroplasmosis (*B. equi*, *B. caballi*, and *Theileria equi*). The 41-element cluster centred on bovine babesiosis, with the use of various approaches such as serology and nested PCR in diagnosis, including phylogenetic analysis. It also highlighted the development of vaccines to control the outbreak of infection in South America, especially Brazil, where live vaccines have been extensively used. The four-element cluster centred on equine piroplasmosis and its etiological agents.

### 3.6. Performance Analysis

#### Top Publishing Journals

Performance analysis in terms of publishing refers to the leading journals with the highest output in a particular research field within a specified period. The top 20 ranked journal with the highest output on babesiosis research is presented in Table 5. A total of 3763 articles on babesiosis were published by 512 journals within the study period. About 10.9% (339) of these articles were published by *Veterinary Parasitology*, which is the top-ranked journal in terms of publications on babesiosis. The chart of the top five journals consists of *Parasitology Research*, *Tick and Tick-Borne Diseases*, *Parasites and Vectors*, and finally *Journal of Veterinary Medical Science*, with 3.7% (142), 3.5% (135), 3.5% (134), and 2.7% (104). 

### 3.7. Funding Analysis 

The funding agencies of babesiosis research were identified within the study period (1982–2022) via funding analysis. This objective was achieved by excluding authors who did not specify the source of funding or those who categorically mentioned in their research that there was no funding. The top 10 leading funding agencies in babesiosis research are presented in Table 6. The United States Department of Health and Human Services was the leading funding agency, with a total of 254 (6.7%) articles, while the National Institute of Health, USA, ranked second, with 238 (6.3%) articles. The Ministry of Education Culture Sports, Science, and Technology, Japan; the Japan Society for the Promotion of Science; and Grants in Aid for Scientific Research completed the top five funding agencies, with 225 (5.9%), 186 (4.9%), and 148 (3.9%) articles, respectively. It is not surprising that the USA, Japan, and some European countries are leading in terms of research and publication on babesiosis; this is associated with funding of such research work.

### 3.8. Strength and Limitations

In this study, bibliometric analysis was conducted using the R tool and its interphase biblioshiny, from 1982 to 2022. To our knowledge, this is the pioneering article focusing on the bibliometric analysis of *Babesia* infection using the R tool across the study period (1982–2022). The present study had some limitations. First, we used a single database for searching the publications in the WoS. However, since the WoS covers more published articles than Scopus and PubMed, a single database search provided more unique datasets than using multiple databases. Moreover, certain institutions/countries’ results might have been overestimated because the WoS counts a document once for each author. Nevertheless, all efforts were made to search from reference sections of the full articles that were accessed for potential articles missed out in the initial search. Additionally, the affiliation address of the original author does not necessarily reflect the country in which the research was carried out or the author of the research project. For collaborative articles, the principal investigator and the source of funding may be in a different country than the primary author. Therefore, we may have missed some documents that do not use informational keywords in the title because we did not search for the terminology used in summaries. Despite these limitations, bibliometric analysis findings such as those presented in this study can be utilised to describe the evolution, patterns, trends, and impact of scientific research across different research fields and disciplines. 

## 4. Conclusions

In this study, we analysed and evaluated the global scientific research output of the literature on *Babesia* infection through data imported via the Web of Science, thus recognising the most relevant authors and the geographical distribution of research inputs and documents. This is the pioneer report of bibliometric analysis on *Babesia* infection using the R tool and its associated interphase of bibliometrix and biblioshiny. The results showed an increase in the number of articles over the last four decades. The findings from this analysis provide a template for identifying hotspots and future trends in the study area. However, this information is not only for active researchers but also for future researchers who have an interest in this field of study. The results of this analysis can be used to describe and visualise research output in this field. This study also helps in identifying the impact of *Babesia* infection research. The results are expected to also spur interest among policymakers at the global level to identify and formulate policies and pool resources that will promote research in *Babesia* infection.

## Figures and Tables

**Table 1:** Presentation of information on babesiosis research extracted from 1982 to 2022.

Description	Frequency/Rates
No. of articles	3763
No. of authors	10,557
Authors of single-authored articles	120
Authors of multi-authored articles	10,437
Article per author	0.356
Author per article	2.81
Co-authors per article	5.52
Keywords plus (ID)	3728
Author keywords (DE)	4559
Collaboration index	2.89
Average citations per article	20.96
References	42,149
**Language**	
English	3596
German	57
Portuguese	36
French	30
Spanish	13
Turkish	9
Hungarian	6
Dutch	5
Russian	5
Polish	4
Italian	2

**Table 2:** Most relevant keyword plus and author keywords on babesiosis studies (1982–2022).

Rank	Keyword Plus (ID)	Frequency (%)	Rank	Author Keywords (DE)	Frequency (%)
1.	Infection	606 (16.1)	1.	Babesiosis	444 (11.7)
2.	Cattle	347 (9.2)	2.	*Babesia*	343 (9.1)
3.	Dogs	289 (7.6)	3.	*Babesia* bovis	299 (7.9)
4.	Identification	282 (7.4)	4.	*Babesia* microti	237 (6.2)
5.	*Bovis*	264 (7.0)	5.	*Babesia* bigemina	199 (5.2)
6.	Prevalence	234 (6.2)	6.	*Babesia* canis	175 (4.6)
6.	Transmission	234 (6.2)	7.	PCR	174 (4.6)
7.	*Plasmodium falciparum*	231 (6.1)	8.	*Babesia gibsoni*	162 (4.3)
8.	Diagnosis	215 (5.7)	9.	Dog	147 (3.9)
9.	Microti	212 (5.6)	10.	Cattle	146 (3.8)
10.	*Bigemina*	211 (5.6)	11.	*Babesia caballi*	105 (2.7)
11.	Ticks	203 (5.3)	12.	Ticks	103 (2.7)
12.	Parasites	193 (5.1)	13.	Elisa	84 (2.2)
13.	PCR	170 (4.5)	14.	*Theileria*	83 (2.2)
14.	Blood	155 (4.1)	15.	*Babesia divergens*	80 (2.1)
15.	Expression	154 (4.0)	16.	Canine babesiosis	75 (1.9)
16.	Antibodies	149 (3.9)	17.	Epidemiology	72 (1.8)
17.	Molecular characterisation	147 (3.9)	18	*Theileria equi*	67 (1.7)
18.	Malaria	141 (3.7)	19	Diagnosis	66 (1.7)
19	*Theileria*	139 (3.6)	20.	*Babesia* spp	62 (1.6)

**Table 3:** Most influential researchers in the field of babesiosis.

Authors	Publications	h-Index	g-Index	First Publication Year
Igarashi I.	231 (6.1%)	28	41	1990
Xuan X.	139 (3.6%)	23	32	1999
Yokoyama N.	130 (3.4%)	23	34	2001
Li Y.	67 (1.7%)	17	23	2008
Yin H.	65 (1.7%)	17	24	2002
Luo J.	57 (1.5%)	17	22	2002
Guan G.	47 (1.2%)	16	22	1998
Baneth G.	45 (1.1%)	25	37	1995
Fujisaki K.	45 (1.1%)	20	31	1991
Brown W.C.	441.1%)	26	39	2002
Liu Z.	40 (1.0%)	15	22	1993
Suarez C.E.	40 (1.0%)	19	32	2015
Liu M.	39 (1.0%)	10	16	2007
Terkawi M.A.	39 (1.0%)	15	24	1991
Knowles D.P.	37 (0.9%)	21	33	1991
Krause P.J.	35(0.9%)	23	33	2008
Liu J.	35 (0.9%)	13	20	2009
Sivakumart T.	35 (0.9%)	14	21	2003
Suzuki H.	35 (0.9%)	13	20	2003
Kumar S.	34 (0.9%)	13	17	1996

**Table 4:** Most productive countries in terms of publications in babesiosis research (1982–2022).

Productivity Based on the Number of Articles						Most Cited Countries	
Rank	Country	Articles (%)	SCP	MCP	MCP Ratio	Country	TC
1.	USA	770 (20.4)	620	150	0.19	USA	24,155
2	Japan	391 (10.3)	195	196	0.50	Japan	6295
3	China	226 (6.0)	183	43	0.19	United kingdom	3209
4	Brazil	217 (5.7)	181	36	0.16	Netherlands	2972
5	Poland	142 (3.7)	107	35	0.24	Germany	2967
6	India	126 (3.3)	121	5	0.03	Spain	2959
7	Germany	114 (3.0)	62	52	0.45	Brazil	2955
8	South Africa	113 (3.0)	65	48	0.42	France	2741
9	France	111 (2.9)	57	54	0.48	Italy	2668
10	Italy	111 (2.9)	60	51	0.45	Australia	2639
11	United Kingdom	105 (2.7)	59	46	0.43	China	2461
12	Australia	90 (2.3)	66	24	0.26	South Africa	2397
13	Spain	89 (2.3)	49	40	0.44	Poland	2335
13	Turkey	89 (2.3)	76	13	0.14	Turkey	1741
14	Israel	61(1.6)	41	20	0.32	Israel	1200
15	Iran	56 (1.4)	54	2	0.03	Switzerland	1193
16	Netherlands	46 (1.2)	20	26	0.56	Portugal	872
16	Portugal	46 (1.2)	12	34	0.73	India	800
17	Argentina	40 (1.0)	21	19	0.47	Hungary	645
18	Switzerland	37 (0.9)	19	18	0.48	Argentina	612

SCP, single-country publication; MCP, multiple-country publication; TC, total citations.

**Table 5:** Top publishing journals on babesiosis research (1982–2022).

Rank	Journal	Frequency (%)
1.	*Veterinary Parasitology*	393 (10.4)
2.	*Parasitology Research*	142 (3.7)
3.	*Tick and Tick-Borne Diseases*	135 (3.5)
4.	*Parasites and Vectors*	134 (3.5)
5.	*Journal of Veterinary Medical Science*	104 (2.7)
6.	*Experimental Parasitology*	91 (2.4)
7.	*International Journal for Parasitology*	76 (2.0)
8.	*Infection and Immunity*	71 (1.8)
8.	*Journal of Clinical Microbiology*	71 (1.8)
8.	*Journal of Parasitology*	71 (1.8)
9.	*Molecular and Biochemical Parasitology*	69 (1.8)
10	*Parasitology*	63 (1.6)
11.	*Transfusion*	57 (1.5)
12.	*Pathogens*	47 (1.2)
12.	*Tropical Animal Health and Production*	47 (1.2)
13.	*Parasitology International*	45 (1.1)
14.	*Annals of the New York Academy of Sciences*	44 (1.1)
15.	*Vector Borne and Zoonotic Diseases*	43 (1.1)
16.	*Indian Veterinary Journal*	37 (0.9)
17.	*Onderstepoort Journal of Veterinary Research*	33 (0.8)

**Table 6:** Top 10 funding agencies on babesiosis research (1982–2022).

Rank	Funding Agencies	Frequency (%)
1.	United States Department of Health and Human Services	254 (6.7)
2.	National Institutes of Health, USA	238 (6.3)
3.	Ministry of Education Culture Sports, Science, and Technology, Japan	225 (5.9)
4.	Japan Society for the Promotion of Science	186 (4.9)
5.	Grants in Aid for Scientific Research	148 (3.9)
6.	National Institute of Allergy Infectious Disease	137 (3.6)
7.	National Natural Science Foundation of China	96 (2.5)
8.	European Commission	64 (1.7)
9.	United States Department of Agriculture	56 (1.4)
10.	National Basic Research Program of China	51 (1.3)

## Data Availability

Data are all available in the manuscript.
